# Aqueous Date Flesh or Pits Extract Attenuates Liver Fibrosis via Suppression of Hepatic Stellate Cell Activation and Reduction of Inflammatory Cytokines, Transforming Growth Factor-***β***1 and Angiogenic Markers in Carbon Tetrachloride-Intoxicated Rats

**DOI:** 10.1155/2015/247357

**Published:** 2015-04-06

**Authors:** Nouf M. Al-Rasheed, Hala A. Attia, Raeesa A. Mohamad, Nawal M. Al-Rasheed, Maha A. Al-Amin, Asma AL-Onazi

**Affiliations:** ^1^Department of Pharmacology and Toxicology, College of Pharmacy, King Saud University, Riyadh 11495, Saudi Arabia; ^2^Biochemistry Department, College of Pharmacy, Mansoura University, Mansoura 35516, Egypt; ^3^Anatomy Department, Faculty of Medicine, King Saud University, Riyadh 11495, Saudi Arabia

## Abstract

Previous data indicated the protective effect of date fruit extract on oxidative damage in rat liver. However, the hepatoprotective effects via other mechanisms have not been investigated. This study was performed to evaluate the antifibrotic effect of date flesh extract (DFE) or date pits extract (DPE) via inactivation of hepatic stellate cells (HSCs), reducing the levels of inflammatory, fibrotic and angiogenic markers. Coffee was used as reference hepatoprotective agent. Liver fibrosis was induced by injection of CCl_4_ (0.4 mL/kg) three times weekly for 8 weeks. DFE, DPE (6 mL/kg), coffee (300 mg/kg), and combination of coffee + DFE and coffee + DPE were given to CCl_4_-intoxicated rats daily for 8 weeks. DFE, DPE, and their combination with coffee attenuated the elevated levels of inflammatory cytokines including tumor necrosis factor-*α*, interleukin-6, and interleukin-1*β*. The increased levels of transforming growth factor-*β*1 and collagen deposition in injured liver were alleviated by both extracts. CCl_4_-induced expression of *α*-smooth muscle actin was suppressed indicating HSCs inactivation. Increased angiogenesis was ameliorated as revealed by reduced levels and expression of vascular endothelial growth factor and CD31. We concluded that DFE or DPE could protect liver via different mechanisms. The combination of coffee with DFE or DPE may enhance its antifibrotic effects.

## 1. Introduction

Chronic liver diseases are a major cause of mortality and morbidity worldwide. Liver fibrosis represents the final common pathway of chronic liver diseases and eventually leads to cirrhosis and its complications including liver failure and hepatocellular carcinoma [1, 2]. Since no curable drug currently exists, the removal of the underlying injurious agents or delay in the progress of liver fibrosis is the most appropriate strategy for patients with a high risk of liver cirrhosis.

Liver fibrosis is characterized by progressive accumulation of extracellular matrix (ECM) proteins, including collagens, resulting in destruction of the hepatic architecture [1, 3]. Among different cell types involved in the deposition of ECM protein, hepatic stellate cells (HSCs) have a predominant role [1]. Under basic conditions, HSCs are quiescent with low mitotic activity and primarily store vitamin A [3]. However, upon liver injury, HSCs are activated via transformation to myofibroblast-like cells that express desmin and *α*-smooth muscle actin (*α*-SMA) [3, 4]. Activated HSCs have the ability to secrete inflammatory and fibrogenic cytokines leading to overproduction of ECM and hepatic inflammation. Accordingly, activation of HSCs is a key step in hepatic fibrosis. Transforming growth factor-*β* (TGF-*β*) is the most potent profibrogenic factor that activates HSCs. Three different isoforms of TGF-*β* (*β*1, *β*2, and *β*3) have been identified, among them TGF-*β*1 is the most extensively studied. In HSCs, TGF-*β*1 favors the transition to myofibroblasts and strongly upregulates production and deposition of ECM [5]. Therefore, strategies aimed at disrupting TGF-*β*1 synthesis with the subsequent inactivation of HSCs markedly decreased fibrosis in experimental models.

Oxidative stress and activation of HSCs result in hepatic inflammation that greatly contributes to liver fibrogenesis. Inflammatory cytokines such as tumor necrosis factor-alpha (TNF-*α*), interleukin-6 (IL-6), and IL-1 *β* further activate HSCs and enhance their proliferation and survival, thus promoting ECM deposition and exacerbating the fibrogenesis [3, 4]. In addition, multiple inflammatory cell interactions with Kupffer cells, platelets, endothelial cells, and hepatocytes mediated by TNF-*α* and IL-6 are involved in the mechanism of fibrogenesis. TNF-*α* induces neutrophil infiltration and stimulates mitochondrial oxidant production in hepatocytes, which are sensitized to undergo apoptosis. In mice deficient for TNF receptors, there is less Kupffer cell activation and reduced collagen deposition compared to wild-type mice implicating the role of TNF-*α* directly in Kupffer cell activation and fibrogenesis [1]. Therefore, suppressing the inflammatory response may help to prevent hepatic fibrosis.

Angiogenesis, the formation of new blood vessels from preexisting ones, plays an important role in the development of liver fibrosis [6]. Expression of proangiogenic factors, particularly, vascular endothelial growth factor (VEGF) significantly increases during the course of liver fibrosis in both clinical [7] and experimental [8–11] studies. The fibrogenic effect of VEGF could be explained through multiple mechanisms including promotion of inflammation and direct effects of VEGF on HSCs [6]. Two tyrosine kinases, fms-like tyrosine kinase (VEGFR-1, flt-1) and the kinase insert domain-containing receptor murine homologue and fetal liver kinase-1 (VEGFR-2, flk-1), both of which are type III tyrosine kinase receptors, have been identified as the main VEGF receptors. By binding with high affinity to these two receptors, VEGF can stimulate endothelial cell proliferation, migration, and differentiation and can induce angiogenesis* in vitro* and* in vivo* [12, 13]. It has been shown that expression of VEGFR-1 and VEGFR-2 was induced during activation of HSC* in vitro* [14]. In experimental liver fibrogenesis, it has been reported that VEGFR-1 expression increased in the liver, and VEGFR-2 was highly expressed [11]. It has been found that inhibition of either VEGFR-1 or VEGFR-2 significantly attenuated liver fibrogenesis accompanied by angiogenesis suppression [6]. CD31 (also known as PECAM-1), a 130 kDa integral membrane protein, is a member of the immunoglobulin superfamily that mediates cell-to-cell adhesion. CD31 mediated endothelial cell-cell interactions are involved in angiogenesis process [15] and play a role in the fibrogenic process. Inhibition of angiogenesis with multitargeted receptor tyrosine kinase inhibitors [16] and blocking the interactions of VEGF with its receptors have been shown to regress or reverse liver fibrosis in experimental animals [6, 17].

According to previous data, a successful reduction in fibrotic tissue could be achieved by alleviating oxidative stress, suppressing HSCs activation, and/or reducing the release of TGF-*β*, inflammatory cytokines, and angiogenic factors such as VEGF, VEGFR-1, and CD31. Recently, several dietary supplements have been reported to have strong antioxidant and anti-inflammatory effects. One of them is date palm fruit (*Phoenix dactylifera, L*) which has great importance from nutritional and economic points of view. Date fruits are composed of a fleshy pericarp and seed (pit) and are very commonly consumed in many parts of the world as a vital component of the diet and a staple food in most of the Arabian countries [18]. The importance of the dates in human nutrition comes from its rich composition of several nutrients in both flesh and pits. These nutrients include carbohydrates, dietary fibers, vitamins, fats, amino acids, protein, and essential minerals [18, 19].

Besides the nutritional value of date palm fruit,* in vitro* [20] and* in vivo* [21–27] studies have demonstrated the potent antioxidant and antimutagenic activities of the date palm fruit extract. Several* in vivo* studies demonstrated that the aqueous and ethanolic extracts of dates were effective in ameliorating the severity of gastric ulceration [27], nephrotoxicity [26], and neurotoxicity [28]. Moreover, the aqueous extract of date fruit exhibited neuroprotective effects in STZ-induced peripheral diabetic neuropathy [29]. The* in vivo* antigenotoxicity of date pits extract has been also evaluated [30].

In addition, Saafi et al. [23], El-Gazzar et al. [24], and El Arem et al. [25] demonstrated a hepatoprotective effect of date palm fruit extract on oxidative damage induced by dimethoate, trichloroacetic, and carbon tetrachloride (CCl_4_), respectively. In addition, aqueous extract of date pits protected the liver from CCl_4_-induced hepatotoxicity via its ability to restore the normal serum markers of liver function [31, 32] and via antioxidant mechanism [31]. Concerning the hepatoprotective effect, only the antioxidant mechanism of date flesh has been previously investigated [21–27], and to our knowledge the antioxidant mechanism of pits was poorly studied [31]. Hepatoprotective efficacy via other mechanisms has not been studied. Being a good source of antioxidant, anti-inflammatory, and anti-angiogenic substances (selenium), we considered undertaking this study to assess the influence of flesh or pits on CCl_4_-induced liver fibrosis in rats. Inactivation of HSCs and suppression of TGF-*β* (the most potent profibrotic factor), TNF-*α*, IL-1*β*, and IL-6 (as inflammatory mediators), and VEGF, VEGFR-1, and CD31 (as angiogenic markers) were taken as target mechanisms.

Coffee has been extensively reported to have beneficial role in protection from liver disease. The consumption of more than three cups of coffee per day has been inversely related to the incidence of nonalcoholic fatty liver disease, fibrosis/cirrhosis, and hepatocellular carcinoma development in subjects with or without hepatitis B and/or C infection [33–36]. Experimental studies have suggested that the intake of instant coffee, conventional coffee, or any of its components can reduce hepatotoxicant-induced liver fibrosis in male rats and mice [10, 37–41]. Mechanistic studies have suggested that coffee and its components are beneficial due to antioxidant properties and HSCs inactivation [38, 39], lowering the concentration of TGF-*β*1, TNF-*α*, and IL-1 in liver tissues [42], and suppression of VEGF expression [10]. Because coffee is usually drunk simultaneously with dates in Arabic countries and according to its beneficial effects on the liver, we used coffee as reference antifibrotic agent in this study and the possible synergistic effects of the combination of coffee with date flesh or pits extract was also investigated.

## 2. Materials and Methods

### 2.1. Reagents and Chemicals

CCl_4_, thiobarbituric acid, Ellman's reagent (5,5′-dithiobis-2-nitrobenzoic acid, DTNB), chloramine-T, and glutathione reductase (GR) kits were purchased from Sigma-Aldrich chemical Co. (St Louis, MO, USA). Date fruits of* Mabroom* variety were obtained from Kingdom Dates Factory in Riyadh, Kingdom of Saudi Arabia. Date pits powder was purchased from a local company in Riyadh, Kingdom of Saudi Arabia. Instant coffee (Nescafe) was obtained from Nestlé (Cheongju, Korea). Commercial kits used for determining albumin and liver enzymes were purchased from Randox Laboratories Ltd. (CRUMLIN, CO. Antrim, UK). Kits used for determining superoxide dismutase (SOD) and glutathione peroxidase (GPx) were obtained from Cayman Chemical Company (USA). ELISA kits for assay of TNF-*α*, IL-6, IL-1*β*, TGF-*β*1, and VEGF were obtained from R&D Co. (Quantikine, R&D systems, Minneapolis, MN, USA). Primary antibodies for *α*-SMA, VEGFR-1, and CD31 immunostaining were obtained from Santa Cruz (Santa Cruz Biotechnology, CA, USA). Primary antibodies for TGF-*β*1 and VEGF immunostaining were purchased from Abcam (Cambridge, UK). Secondary antibody was obtained from Sigma-Aldrich. All other reagents were of analytical quality.

### 2.2. Preparation of Date Flesh Extract

The date flesh was manually separated from the pits and soaked in cold distilled water (1 : 3 ratio, g/mL) and kept for 48 hours in a refrigerator (4°C) with continuous stirring. The extract was filtered and the aqueous supernatant was then used [32]. Aqueous extract was selected because most of the antioxidants and active components in dates are extracted in water [21].

### 2.3. Preparation of Date Pits Extract

The dried pit powder was soaked with water (1 : 10 ratio g/mL) under agitation at 4°C for 48 hrs. After 48 h, the extract was filtered and the aqueous supernatant was then used. During the experiment, the aqueous date flesh extract (DFE) and date pit extract (DPE) were daily prepared and administrated to rats by oral gavage.

### 2.4. Dose-Response Experiment for Date Flesh and Pits

The dose of 4 mL/kg/day was used previously [21, 23, 25, 32] as an effective dose of both date flesh and pits. In the current study, a preliminary experiment with histological endpoints was carried out with four concentrations (2, 4, 6, and 8 mL/kg) of date flesh and pits in CCl_4_-treated rats for the selection of the most appropriate and effective dose of both date flesh and pits. Forty rats were divided into ten groups (4 rats each) and treated by oral gavage for 8 weeks as follows.

 Group (1): normal control with no treatment.
 Group (2): control treated with CCl_4_ only.
 Groups (3), (4), (5), and (6): CCl_4_ treated with aqueous extract of date flesh at 2, 4, 6, and 8 mL/kg/day, respectively.
 Groups (7), (8), (9), and (10): CCl_4_ treated with aqueous extract of date pits at 2, 4, 6, and 8 mL/kg/day, respectively.

At the end of the 8 weeks, histological endpoints were studied. Small blocks of tissues were embedded in paraffin wax and then cut into 4 *μ* sections by microtome. Sections were stained with Masson's trichome to detect collagen deposition.

### 2.5. Induction of Liver Fibrosis and Study Design with the Selected Dose of Date Flesh and Pits

Seventy rats were enrolled in the study. One week after acclimatization, rats were randomly divided into seven groups of ten rats each as follows.

 Group I: normal, control with no treatment.
 Group II: control treated with CCl_4_ only.
 Group III: CCl_4_ treated with coffee.
 Group IV: CCl_4_ treated with aqueous extract of date flesh (DFE).
 Group V: CCl_4_ treated with aqueous extract of date pits (DPE).
 Group VI: CCl_4_ treated with combination of coffee + DFE.
 Group VII: CCl_4_ treated with combination of coffee + DPE.

Liver fibrosis was induced in all groups, except for Group I, by intraperitoneal injection of 0.4 mL/kg of 40% CCl_4_ in corn oil three times weekly for 8 weeks. DFE and DPE were administered by oral gavage daily for 8 weeks. The administered dose was 6 mL/kg/day (the most effective dose from the dose response study). Coffee was dissolved in hot water and given at a dose 300 mg/kg/day [10] by oral gavage for 8 weeks. The dose of coffee was chosen considering four standard cups as the amount of drinking daily.

### 2.6. Serum and Liver Tissue Processing

After overnight fasting, at the end of 8 weeks, all rats were anesthetized and sacrificed by decapitation. Blood was collected, allowed to coagulate, and centrifuged at 4000 rpm for 15 minutes. Sera were then divided into aliquots to be used for determination of albumin and liver enzymes.

### 2.7. Preparation of Liver Tissue Homogenate

The livers were removed and washed from excess blood with saline. A part of each liver was cut with scissors, weighed, and homogenized in phosphate buffered saline (PBS; NaCl 8 g/L, KCl 0.2 g/L, Na_2_HPO_4_ 144 g/L and KH_2_PO_4_ 0.24 g/L, pH 7.4) to prepare 20% homogenate. Five mL of PBS was added per one g liver and homogenized in Ultra Turrax (IKA-USA) for 40 seconds at 4°C. The homogenates were centrifuged at 3000 rpm for 10 min at 4°C and the supernatant was taken for the assay of oxidative stress markers, TNF-*α*, IL-6, IL-1*β*, TGF-*β*, and VEGF. For histological and immunostaining of *α*-SMA and CD31, samples of the right lobe of the liver were fixed with 4% buffered formalin in PBS (pH 7.4) for at least 24 hrs, dehydrated in ascending graded ethanol, and then embedded in paraffin wax.

### 2.8. Assessment of Liver Function

Serum activities of liver enzymes, alanine aminotransferase (ALT), aspartate aminotransferase (AST), lactate dehydrogenase (LDH), alkaline phosphatase (ALP), and gamma glutamyl transferase (GGT) as well as albumin levels were measured with routine laboratory methods using commercially available kits from Randox Co.

### 2.9. Assessment of Oxidative Stress Markers

#### 2.9.1. Assay of Lipid Peroxidation

The process of lipid peroxidation results in the formation of malondialdehyde (MDA) as a later product in the sequence of the oxidation reactions. The thiobarbituric acid (TBA) assay was used to assess the MDA concentrations as described by Ohkawa et al. [43]. Briefly, a mixture of 0.5 mL of 0.6% TBA, 1.25 mL of 20% trichloroacetic acid (TCA), and 250 *μ*L of liver homogenate or MDA standards was incubated at 100°C for 60 min. The mixture was then cooled and centrifuged for 10 min at 4°C. The absorbance of the developed pink-colored product was measured at 535 nm against a reagent blank. 1,1,3,3-Tetraethoxypropane, a form of MDA, was used as standard in this assay.

#### 2.9.2. Assay of Reduced Glutathione (GSH)

GSH constitutes the first line of defense against free radicals and is a critical determinant of tissue susceptibility to oxidative damage. Total GSH was determined according to the method described by Moron et al. [44] based on the reduction of 5,5′-dithiobis-(2-nitrobenzoic acid) by sulfhydryl groups to form 2-nitro-5-mercaptobenzoic acid, which has an intense yellow color. A sample of hepatic homogenate was mixed with equal volume of 25% TCA and then centrifuged at 4°C at 3000 rpm for 10 min. 0.5 mL of supernatant or GSH standard was then added to 4.5 mL of Ellman's reagent and the produced yellow color was measured at 412 nm.

#### 2.9.3. Superoxide Dismutase (SOD), Glutathione Reductase (GR), and Glutathione Peroxidase (GPx) Assay

Activities of SOD, GPx, and GR in the liver tissue were determined using assay kits according to the manufacturer's protocol.

### 2.10. ELISA Assay of Proinflammatory Markers

Hepatic levels of TNF-*α*, IL-6, and IL-1*β* were determined using Quantikine Immunoassay kits (R&D Systems) according to the manufacturer's instructions.

### 2.11. Measurement of Fibrogenic Markers (Hydroxy Proline, TGF-*β*, and *α*-SMA)

#### 2.11.1. Determination of Hydroxyproline (Hyp) Content in Hepatic Tissue

The Hyp content of the liver was used as an indirect measure of tissue collagen content and was expressed as (mg/g dry weight). Hyp was determined according to a modified method by Jamall et al. [45]. 100 mg of liver tissue was homogenized and hydrolyzed in HCl at 110°C for 18 h. The hydrolysate was filtrated, and chloramine-T was added to a final concentration of 2.5 mM. The mixture was then treated with 410 mM p-dimethyl-amino-benzaldehyde and incubated for 30 min at 60°C. Finally, the absorbance of samples was read at 560 nm against reagent blank.

#### 2.11.2. ELISA Assay of TGF-*β*1 (The Most Potent Profibrogenic Factor)

Levels of TGF-*β*1 in hepatic tissues were determined using Quantikine Immunoassay kits (R&D Systems) according to the manufacturer's instructions.

#### 2.11.3. Immunostaining of TGF-*β*1 and *α*-SMA

Immunostaining of paraffin sections of the liver was performed for detection of TGF-*β*1 and vascular *α*-SMA (the hallmark of HSCs activation). The primary antibodies, mouse monoclonal anti-rat *α*-SMA (sc-130617), and rabbit monoclonal anti-rat TGF-*β*1 (ab169771) were used for detection. ImmunoCruz ABC staining system from Santa Cruz was used for both processes. The procedure involved the following steps: endogenous peroxidase activity was inhibited by 3% H_2_O_2_ in distilled water for 5 minutes, and then the sections were washed in Tris buffered saline (TRS) (Sigma, T 5030-100 TAB, PH 7.6) for 10 minutes. Nonspecific binding of antibodies was blocked by incubation with protein block for 5 minutes. Sections were incubated with anti-rat *α*-SMA (diluted 1 : 200) and with anti-TGF-*β*1 (diluted 1 : 100) for 1 hour at room temperature. Sections were washed in Tris buffer for 3 times each for 3 minutes and then incubated with biotinylated anti-rabbit IgG for 30 minutes. This was followed by washing in Tris buffer for 3 times, each for 3 minutes. Peroxidase was detected with working solution of Diaminobenzidine (DAB) substrate for 10 minutes. Finally sections were washed in distilled water for 10 minutes, nuclei were stained with Mayer's hematoxylin, and sections were mounted in DPX. For negative control sections, the same procedure was followed with omission of incubation in the primary antibody.

### 2.12. Measurement of Angiogenic Markers (VEGF as Potent Angiogenic Marker, VEGFR-1, and CD31 as Marker for Endothelial Cells)

#### 2.12.1. ELISA Assay of VEGF

Hepatic levels of VEGF were measured using Quantikine Immunoassay kits (R&D Systems) according to the manufacturer's instructions.

#### 2.12.2. Immunostaining for CD31 (PECAM), VEGF, and VEGFR-1

The procedure was the same as that of *α*-SMA with using the antibodies, goat polyclonal anti-rat PECAM (sc-1506, 1 : 100 dilution), mouse monoclonal anti-rat VEGF (ab1316, 1 : 100 dilution), and rabbit polyclonal anti-rat VEGFR-1 (sc-9029, 1 : 100 dilution).

### 2.13. Histological Examination

The excised liver tissues were fixed in 4% buffered formalin at 4°C for 24 hours and processed to prepare 5-*μ*m-thick paraffin sections. These sections were stained with hematoxylin and eosin (H&E) to study the morphological changes and with Masson's trichrome stain to detect collagen fibers.

### 2.14. Statistical Analysis

Data were expressed as means ± SEM. Statistical comparisons were performed using Prism GraphPad software version 4 (San Diego, California, USA) using one way ANOVA followed by Tukey-Kramer post hoc test. *P* values < 0.05 were considered statistically significant.

## 3. Results

### 3.1. Preliminary Study for Selection of the Most Appropriate Dose of DFE and DPE

**Figure 1:**
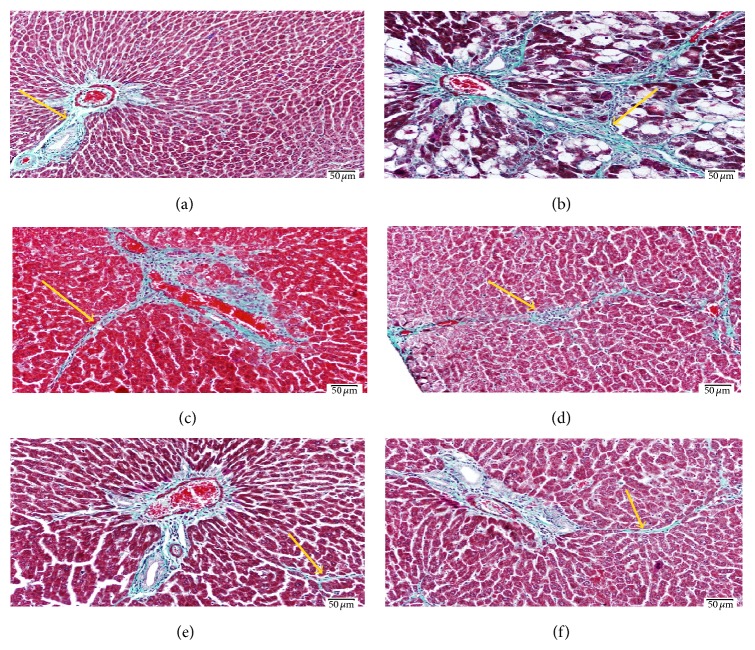
Light photomicrograph of liver sections stained with Masson's trichrome to demonstrate the fibrous tissue (scale bar 50 *μ*m). (a) Control liver showing normal amount and distribution of fibrous tissue (arrow) mainly in the portal area. (b) Liver section from rat exposed to CCl_4_ showing increase of the fibrous tissue (arrow) which extends outside the portal area. (c) Liver from rat exposed to CCl_4_ and receiving 2 mL/kg of DFE showing mild decrease of the amount abnormal deposited fibrous tissue. Also (d) represents liver from rat exposed to CCl_4_ and 4 mL/kg of DFE and shows decrease of fibrous tissue but still abnormally deposited outside portal area. ((e), (f)) Liver sections from rat exposed to CCl_4_ and receiving 6 and 8 mL/kg of DFE, respectively, show apparently normal amount and distribution of fibrous tissue if compared with the control group (a).

**Figure 2:**
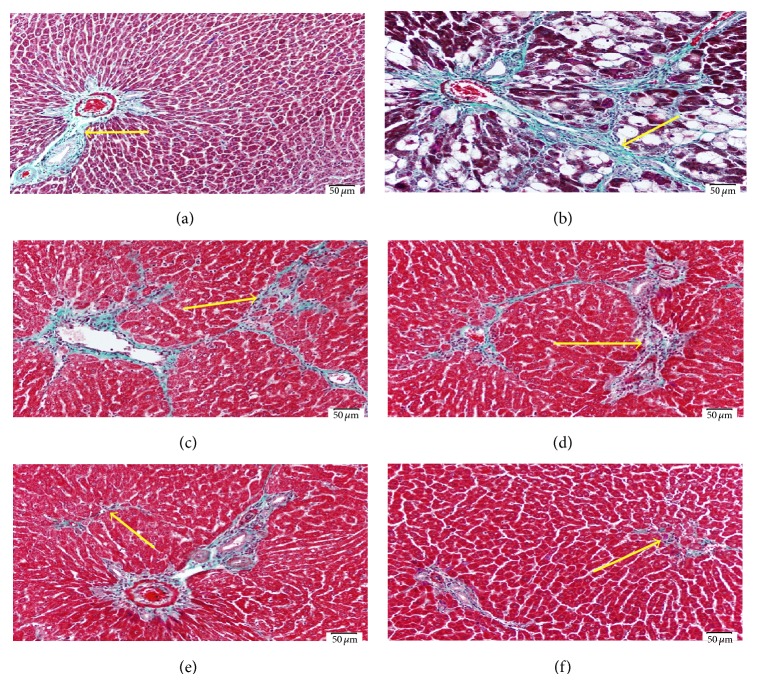
Light photomicrographs of liver sections stained with Masson are trichrome to demonstrate the fibrous tissue (scale bar 50 *μ*m). (a) Control liver showing normal amount and distribution of fibrous tissue (arrow) mainly in the portal area. (b) Liver section from rat exposed to CCl_4_ showing increase of the fibrous tissue (arrow) which extends outside the portal area. (c) Liver from rat exposed to CCl_4_ and receiving 2 mL/kg of DPE showing mild decrease of the amount abnormal deposited fibrous tissue. Also (d) represents liver from rat exposed to CCl_4_ and 4 mL/kg of DPE and shows decrease of fibrous tissue but still abnormally deposited outside portal area. ((e), (f)) Liver sections from rat exposed to CCl_4_ and receiving doses of 6 and 8 mL/kg of DPE, respectively, show apparently normal amount and distribution of fibrous tissue if compared with the control group (a).

As indicated by Masson trichome staining (Figures 1 and 2), the dose 2 mL/kg of both DFE and DPE did not show prominent decrease in collagen deposition. Doses 4, 6, and 8 showed a prominent decrease in collagen content; however, the doses 6 and 8 mL/kg of DFE (Figure 1) and DPE (Figure 2) showed the best reduction in the amount of collagen deposition compared to dose 4 mL/kg and so supposed to exhibit the best protection from CCl_4_-induced liver fibrosis. As the effect of 6 and 8 mL/kg were almost the same and both showed apparently normal amount and distribution of fibrous tissue if compared with the normal control group so, we selected the dose 6 mL/kg as the most appropriate dose to complete the main study. Liver function was also assessed by determining ALT activity (data not shown) that revealed that doses 6 and 8 mL/kg have the best improvement in ALT activity compared to CCl_4_-intoxicated rats.

### 3.2. Effect of Coffee, DFE, DPE, and the Combination Groups on Liver Enzymes and Albumin Levels in CCl_4_-Intoxicated Rats

Biochemical data showed that CCl_4_-treated rats induced a pronounced increase in the serum activities of ALT, AST, GGT, and ALP (*P* < 0.001) and in the activities of LDH (*P* < 0.01) compared to normal control rats (Table 1). Serum levels of albumin were significantly reduced (*P* < 0.001). The elevated activities of ALT and LDH were effectively attenuated by coffee, DFE, DPE, and the combination treatments (*P* < 0.001). The activities of AST were significantly lowered by DFE and its combination with coffee (*P* < 0.01) and to lesser extent by coffee, DPE, and their combination (*P* < 0.05) compared to CCl_4_-treated rats. The activities of ALP and GGT were significantly lowered by treatment with DFE, DPE, and their combination with coffee (*P* < 0.001) and by treatment with coffee alone (*P* < 0.05). The activities of ALT, ALP, and GGT were significantly improved by DFE alone and by DFE + coffee (*P* < 0.01) compared to coffee alone (*P* < 0.05). Moreover, the combination of coffee + DPE significantly lowered ALT activities compared to coffee alone (*P* < 0.05). All groups (coffee, DFE, DPE, and the combination groups) significantly ameliorated the reduction in serum albumin levels (*P* < 0.001) with no significant differences between the combination groups with individual treatments.

### 3.3. Effect of Coffee, DFE, DPE, and the Combination Groups on Oxidative Stress Markers in CCl_4_-Intoxicated Rats

**Figure 3:**
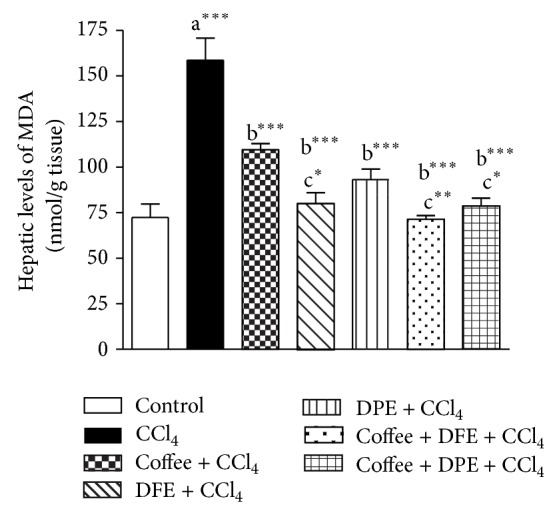
Effect of coffee, date flesh extract (DFE), date pits extract (DPE), and the combination groups on hepatic levels of MDA, a marker of lipid peroxidation in CCl_4_-intoxicated rats. Values are expressed as mean ± SEM. a: significantly different from normal control group, b: significantly different from CCl_4_-treated group; c: significantly different from coffee-treated group, ^∗∗∗^ *P* < 0.001, ^∗∗^ *P* < 0.01, ^∗^ *P* < 0.05.

**Figure 4:**
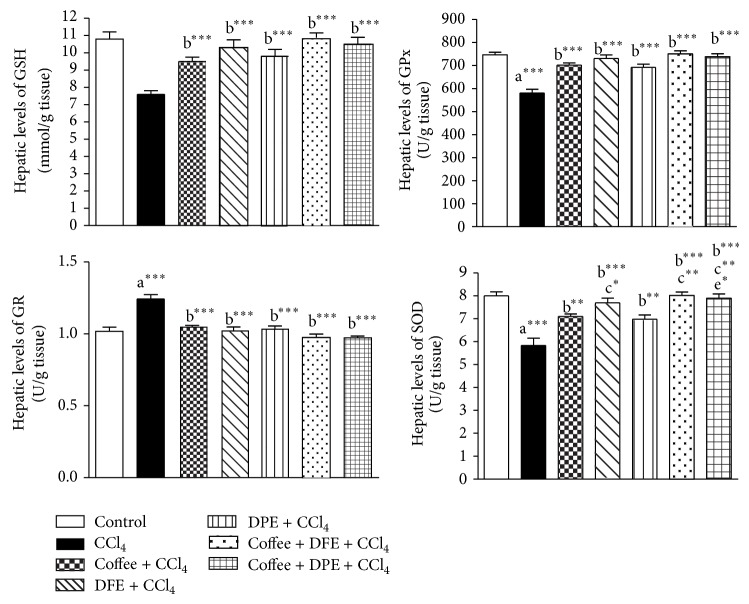
Effect of coffee, date flesh extract (DFE), date pits extract (DPE), and the combination groups on hepatic levels of reduced glutathione (GSH), glutathione reductase (GR), glutathione peroxidase (GPx) and superoxide dismutase (SOD) activities in CCl_4_-intoxicated rats. Values are expressed as mean ± SEM. a: significantly different from normal control group; b: significantly different from CCl_4_-treated group; c: significantly different from coffee-treated group; e: significantly different from DPE-treated group. ^∗∗∗^ *P* < 0.001, ^∗∗^ *P* < 0.01, ^∗^ *P* < 0.05.

CCl_4_ induced excessive lipid peroxidation in hepatic tissues as revealed by the drastically elevated hepatic levels of MDA, an end product of lipid peroxidation (*P* < 0.001) compared to normal control rats (Figures 3 and 4). The levels of GSH, a nonenzymatic antioxidant, and the activities of SOD and GPx, the enzymatic antioxidant, were significantly reduced with CCl_4_ treatment (*P* < 0.001) compared to normal control. On the other hand, the activity of GR was significantly higher in CCl_4_-intoxicated rats (*P* < 0.001). Coffee, DFE, DPE, and the combination groups significantly ameliorated the CCl_4_-induced oxidative stress in hepatic tissue as revealed by the significantly lowered levels of MDA and GR (*P* < 0.001) together with the significant increase in hepatic levels of GSH (*P* < 0.001), GPx (*P* < 0.001), and SOD. SOD activities were increased significantly by coffee, DPE (*P* < 0.01), and more effectively DFE alone and by the combination groups (*P* < 0.001) compared to CCl_4_-intoxicated rats.

The high MDA levels were significantly alleviated by DFE alone (*P* < 0.05) and by the combination of DFE + coffee (*P* < 0.01) and DPE + coffee (*P* < 0.05) compared to coffee-treated group. SOD activities were significantly raised in rats treated with DFE alone (*P* < 0.05), DFE + coffee (*P* < 0.01), and DPE + coffee (*P* < 0.01) compared to coffee alone. SOD was also significantly higher in DPE + coffee compared to DPE alone (*P* < 0.05).

### 3.4. Effect of Coffee, DFE, DPE, and the Combination Groups on the Inflammatory Markers TNF-*α*, IL-1*β*, and IL-6 in CCl_4_-Intoxicated Rats

**Figure 5:**
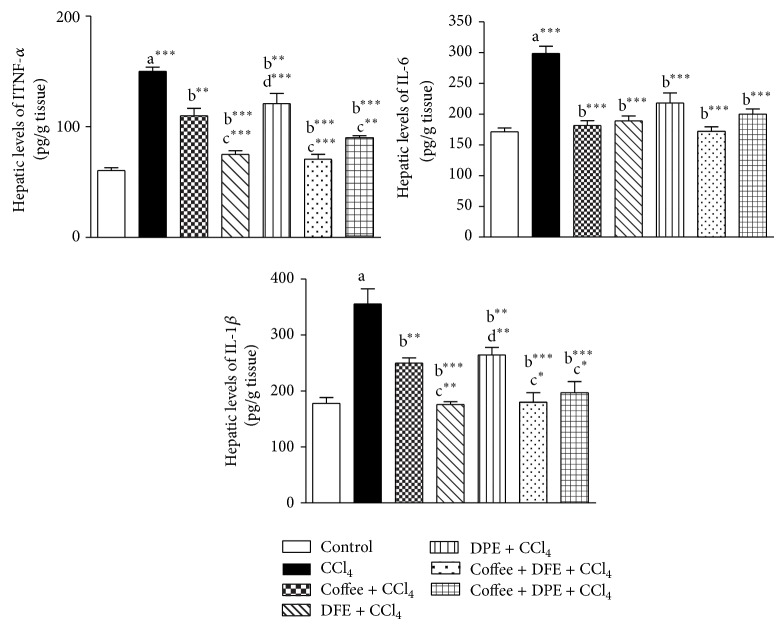
Effect of date flesh extract (DFE), date pits extract (DPE), coffee, and the combination groups on hepatic levels of proinflammatory mediators. (a) Tumor necrosis factor *α* (TNF-*α*), (b) interleukin-6 (IL-6), and (c) interleukin-1*β* (IL-1*β*) in CCl_4_-intoxicated rats. Values are expressed as mean ± SEM. a: significantly different from normal control group; b: significantly different from CCl_4_-treated group; c: significantly different from coffee-treated group; d: significantly different from DFE-treated group. ^∗∗∗^ *P* < 0.001, ^∗∗^ *P* < 0.01, ^∗^ *P* < 0.05.

CCl_4_-intoxicated rats expressed significantly higher hepatic levels of proinflammatory cytokines (*P* < 0.001) including TNF-*α*, IL-1*β*, and IL-6, which have been shown to play important roles in the development of the fibrosis. However, remarkable decreases in the levels of all those three parameters were observed by the simultaneous administration of DFE and the combination of coffee + DFE and coffee + DPE (*P* < 0.001) and by DPE and coffee (*P* < 0.01) compared to CCl_4_-intoxicated rats. It was noted that DFE alone and the combination of coffee + DFE and coffee + DPE significantly improved the hepatic levels of TNF-*α* and IL-1*β* levels compared to coffee alone (Figures 5(a) and 5(c)); however, no significant differences were observed in the case of IL-6 (Figure 5(b)). In addition, the levels of TNF-*α* and IL-1*β* were significantly lowered by DFE alone compared to DPE alone (*P* < 0.001 and *P* < 0.01, resp.).

### 3.5. Effect of Coffee, DFE, DPE, and the Combination Groups on Fibrotic Markers, Collagen Deposition, TGF-*β*1, and *α*-SMA in CCl_4_-Intoxicated Rats (Figures 6(a)–6(e))

#### 3.5.1. The Effect on Collagen Deposition (Figures 6(a) and 6(b))

**Figure 6:**
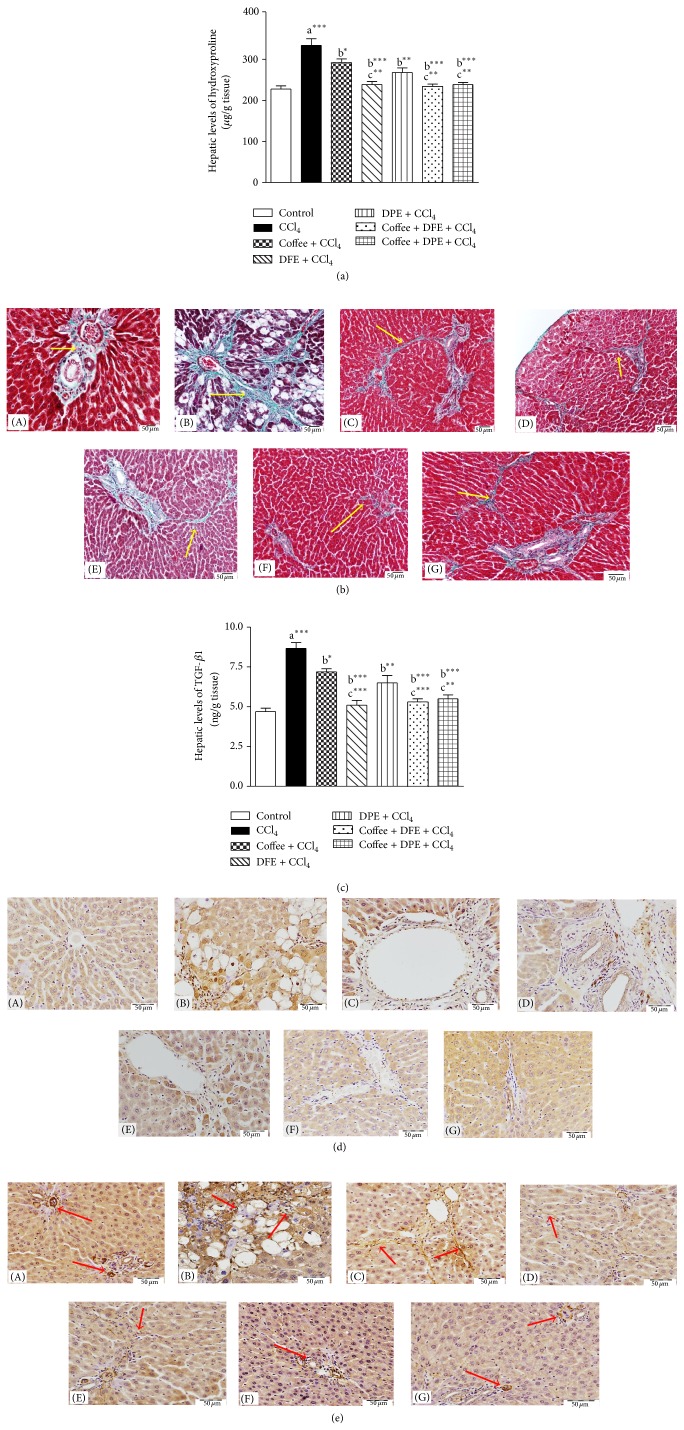
(a) Effect of date flesh extract (DFE), date pits extract (DPE), coffee and the combination groups on hydroxyproline content in hepatic tissue of CCl_4_-intoxicated rats. Values are expressed as mean ± SEM. a: significantly different from normal control group; b: significantly different from CCl_4_-treated group. ^∗∗∗^ *P* < 0.001, ^∗∗^ *P* < 0.01, ^∗^ *P* < 0.05. (b) Light microscopic photomicrographs of liver tissue stained with Masson's trichrome stain (scale bar = 50 *μ*m). (A) Control liver showing normal hepatic fibrous tissue distribution restricted mainly to the portal area, while demarcation between classic hepatic lobule is observed due to very delicate fibrous tissue. (B) Represent liver section of rat receiving CCl_4_ showing marked increase of fibrous tissue arranged in irregular way between degenerated cells and sinusoids, causing destruction of classic architecture of hepatic lobules. Liver sections from rats treated with coffee (C), DFE (D), DPE (E), and the combination of coffee + DFE (F) and coffee + DPE (G) showing marked decrease of abnormally extra deposited fibrous tissue. The improvement is prominent in DFE than coffee alone or DPE alone. Sections from rats receiving combination showed apparently normal amount and distribution of fibrous tissue. (c) Effect of date flesh extract (DFE), date pits extract (DPE), coffee, and the combination groups on hepatic levels of transforming growth factor beta-1 (TGF-*β*1) in CCl_4_-intoxicated rats. Values are expressed as mean ± SEM. a: significantly different from normal control group; b: significantly different from CCl_4_-treated group; c: significantly different from coffee-treated group. ^∗∗∗^ *P* < 0.001, ^∗∗^ *P* < 0.01, ^∗^ *P* < 0.05. (d) Light microscopic photomicrographs of liver tissue immunostained with primary anti-TGF-*β*1 antibody (scale bar = 50 *μ*m). (A) Control liver showing normal very weak immune reactivity of hepatocytes cytoplasm while the nuclei are not stained, and also the endothelial cells of both central vein and hepatic sinusoids are not stained. (B) represents liver section of rat receiving CCl_4_ showing strong abnormal immune reactivity of most of hepatocytes' cytoplasm and nuclei, and also most of cells of structures of portal area show strong nuclear immune reactivity. Panels (C), (D), (E), (F), and (G) represent liver sections from rat receiving coffee, DFE, DPE, coffee + DFE, and coffee + DPE, respectively, showing few hepatocytes with strong ((C), (E)) or moderate (D) immune reactivity especially those around portal area. In (F) and (G) hepatocytes' cytoplasm and few cells of structures of portal areas show weak positivity especially in rats receiving a combination of coffee + DFE (F). (e) Light microscopic photomicrographs of liver tissue immunostained with primary anti-*α*-SMA antibody (scale bar = 50 *μ*m). (A) Control liver showing normal positive immune reactivity of smooth muscle of the blood vessels of the portal area without immune staining positivity in between hepatocytes, while (B) represents liver section of rat receiving CCl_4_ in which strong abnormal distributed immune reactivity, especially around degenerated hepatocytes, is prominent. Panels (C), (D), (E), (F), and (G) represent liver sections from rat receiving coffee, DFE, DPE, coffee + DFE, and coffee + DPE, respectively, showing marked decrease of the immune reactivity outside portal areas especially in groups receiving DFE and the combination.

The contents of hydroxyproline (Hyp) in liver tissue were measured as a collagenic biomarker. In addition, Masson trichome staining was performed to assess the collagen deposition in hepatic tissue. We found that CCl_4_-treated rats showed significantly enhanced Hyp expressions (*P* < 0.001) (Figure 6(a)); however, the elevated Hyp levels were notably reduced by DFE alone and by the combination treatments (*P* < 0.001) followed by DPE (*P* < 0.01) and coffee (*P* < 0.05). The Hyp content was significantly lower in the combination of DFE + coffee and DPE + coffee and in DFE-treated rats compared to coffee alone (*P* < 0.01). Liver sections stained with Masson trichrome (Figure 6(b)) showed also high irregular deposition of fibrous tissue in CCl_4_-intoxicated rats, while treated groups showed marked decrease of abnormal fibrous tissue specially in groups receiving DFE alone and the combinations of coffee + DFE and coffee + DPE which showed apparently normal amount and distribution of fibrous tissues restricted to the portal areas.

#### 3.5.2. The Effect on Hepatic Levels and Expression of TGF-*β*1 (Figures 6(c) and 6(d))

Among fibrotic factors, TGF-*β*1 plays an important role in accumulation of ECM. CCl_4_ caused a highly significant increase in concentration of TGF-*β*1 in liver tissue (Figure 6(c)) compared to normal control (*P* < 0.001). Treatment with coffee, DFE, DPE, and the combination groups significantly suppressed the concentration of TGF-*β*1 compared to CCl_4_-treated rats. The best improvement was achieved by DFE alone and the combination of DFE + coffee and DPE + coffee (*P* < 0.001) followed by DPE alone (*P* < 0.01) and coffee (*P* < 0.05). DFE alone and the combination of DFE + coffee and DPE + coffee showed significant reduction in TGF-*β*1 compared to coffee alone (*P* < 0.001, *P* < 0.01). These results were supported by the immunostaining of sections from liver tissue for the detection of TGF-*β*1 (Figure 6(d)). Immunohistochemical investigation showed strong and massive immunopositivity in hepatocytes' cytoplasm and nuclei in the group treated with CCl_4_ only, while rats receiving coffee, DFE, DPE, and the combination showed marked decrease of immune reactivity where only few hepatocytes revealed strong (Coffee and DPE), moderate (DFE), or weak immune reactivity (the combination groups) especially those around portal area.

#### 3.5.3. The Effect on Hepatic Expression of *α*-SMA, the Hallmark of HSCs Activation (Figure 6(e))

Immunostained section of liver tissues for detection of *α*-SMA fibers (Figure 6(e)) showed strong and massive immunopositivity especially around degenerated hepatocytes in CCl_4_-treated group only, while rats receiving coffee, DFE, DPE, and the combination treatment showed marked decrease of immune reactivity specially in those receiving DFE and the combination of coffee + DFE or coffee + DPE in which the immune reactivity is restricted to the vessels of the portal areas similar to that of control group. The improvement was prominent with DFE and combination groups.

### 3.6. Effect of Coffee, DFE, DPE, and the Combination Groups on the Angiogenic Markers VEGF, VEGFR-1, and CD31 (PECAM) in CCl_4_-Intoxicated Rats (Figures 7(a)–7(d))

**Figure 7:**
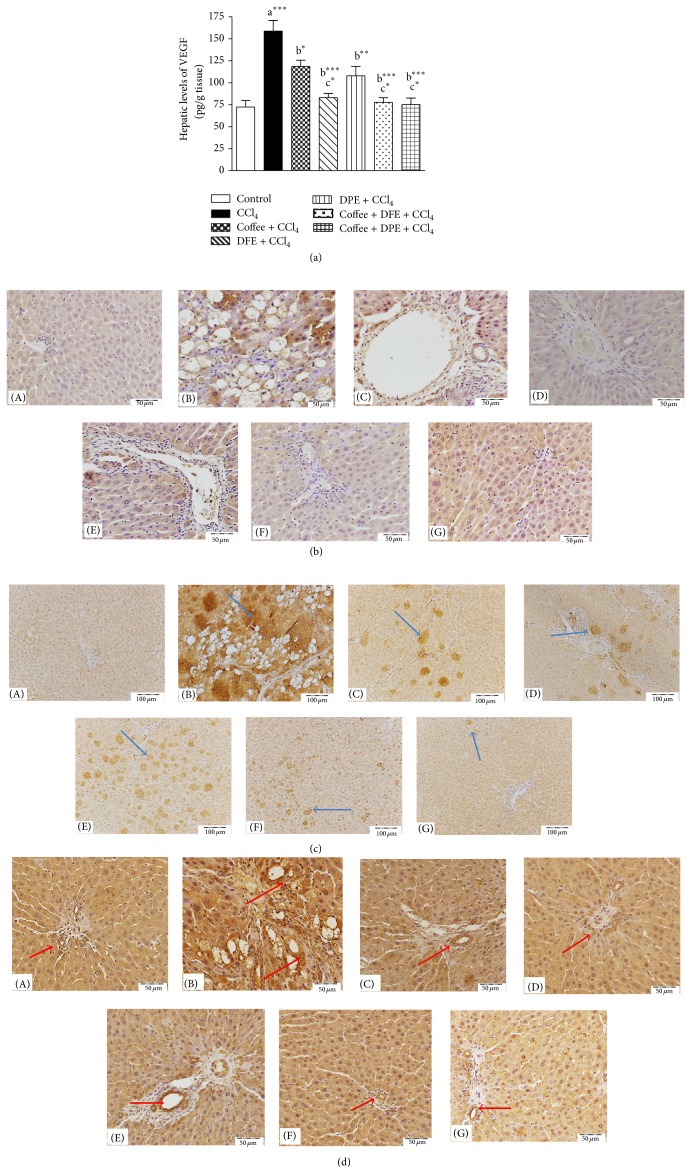
(a) Effect of date flesh extract (DFE), date pits extract (DPE), coffee, and the combination groups on hepatic levels of vascular endothelial growth factor (VEGF) in CCl_4_-intoxicated rats. Values are expressed as mean ± SEM. a: significantly different from normal control group; b: significantly different from CCl_4_-treated group; c: significantly different from coffee-treated group. ^∗∗∗^ *P* < 0.001, ^∗∗^ *P* < 0.01, ^∗^ *P* < 0.05. (b) Light microscopic photomicrographs of liver tissue immunostained with primary anti-VGEF antibody (scale bar = 50 *μ*m). (A) Control liver showing negative immune reactivity of hepatocytes, while (B) represents liver section of rat receiving CCl_4_ showing strong abnormal irregularly distributed immune reactivity, especially between degenerated hepatocytes' cytoplasm and nuclei. Panels (C), (D), (E), (F), and (G) that represent liver sections from rat receiving coffee, DFE, DPE, coffee + DFE, and coffee + DPE, respectively, showed marked decrease in the intensity of the immune reactivity of hepatocytes surrounding portal areas especially in rats treated with the DFE and the combination of coffee + DFE. (c) Light microscopic photomicrographs of liver tissue immunostained with anti-VEGFR-1 primary antibody (scale bar = 100 *μ*m). (A) Control liver showing normal few weak immune positive cellular and nuclear receptors. (B) represents liver section of rat receiving CCl_4_ in which there are focal strong abnormal immune reactivity of most of hepatocytes' cell membrane and nuclei. Panels (C), (D), (E), (F), and (G) that represent liver sections from rat receiving coffee, DFE, DPE, coffee + DFE, and coffee + DPE, respectively, revealed a decrease in the number of immunostained hepatocytes with marked depletion of immune positivity observed in rats treated with coffee + DPE and then with coffee + DFE and DFE alone-treated groups. (d) Light microscopic photomicrographs of liver tissue immunostained with primary anti-PECAM (CD31) antibody (scale bar = 50 *μ*m). (A) Control liver showing normal positive immune reactivity of endothelial cells cell membranes of the blood vessels of the portal area and blood sinusoids, while (B) represents liver section of rat receiving CCl_4_ in which strong abnormal irregularly distributed immune reactivity, especially around degenerated hepatocytes, is prominent. Panels (C), (D), (E), (F), and (G) that represent liver sections from rat treated with coffee, DFE, DPE, coffee + DFE, and coffee + DPE, respectively, showed marked decrease of the immune reactivity outside portal areas especially in groups receiving DFE and combination.

The results obtained from this study revealed that CCl_4_ induced a significant elevation in hepatic levels of VEGF (*P* < 0.001, Figure 7(a)). Coffee, DFE, DPE, and the combination groups significantly attenuated these high levels compared to CCl_4_-treated rats. The most prominent improvement was seen with DFE alone, coffee + DFE, and coffee + DPE (*P* < 0.001) followed by DPE alone (*P* < 0.01) and coffee (*P* < 0.05). The combination of coffee + DFE and coffee + DPE exhibited significantly reduced VEGF levels compared to coffee alone (*P* < 0.05). Immunohistochemical study of liver tissue for the detection of VEGF (Figure 7(b)) and VEGFR-1 (Figure 7(c)) showed strong and massive immunopositivity in the group treated with CCl_4_ only. Immunostaining for detection of VEGF showed abnormal irregularly distributed immune reactivity, especially between degenerated hepatocytes' cytoplasm and nuclei. Immunostaining for detection of VEGFR-1 revealed focal strong abnormal immune reactivity of most of hepatocytes' cell membrane and nuclei. Rats receiving coffee, DFE, DPE, and the combination showed marked decrease of this immune reactivity specially in those receiving the combination of coffee + DFE, coffee + DPE, and DFE alone. Figure 7(d) revealed that the liver section from rat receiving CCl_4_ showed strong abnormal irregularly distributed immune reactivity of CD31, especially around degenerated hepatocytes. However, liver sections from rats treated with coffee, DFE, DPE, coffee + DFE, and coffee + DPE showed marked decrease of the immune reactivity outside portal areas especially in groups receiving DFE and the combination.

### 3.7. Histopathological Examination by H&E Staining

**Figure 8:**
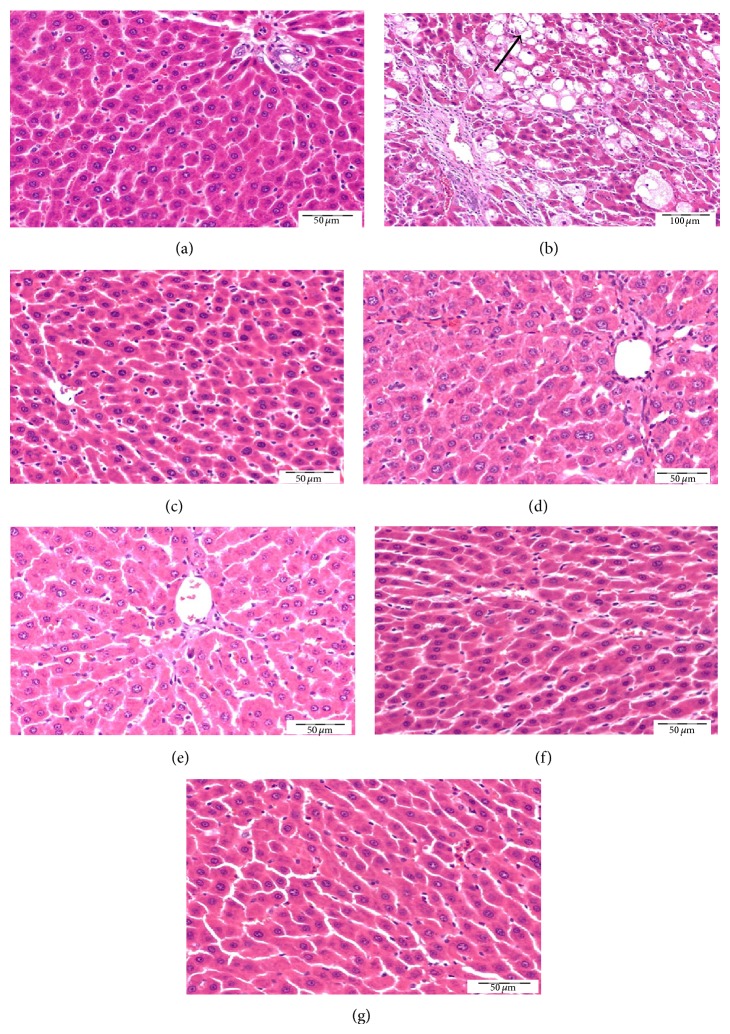
Light microscopic photomicrographs of liver tissue stained H&E (scale bar = 50 *μ*m except B = 100 *μ*m). (a) Control liver showing normal hepatic architecture, normal hepatocytes, and blood sinusoids, while (b) represents liver section of rat receiving CCl_4_ in which most of hepatocytes are degenerated and swollen with vacuolated cytoplasm and pyknotic nuclei. There is complete loss of hepatic lobule architecture. Panels (c), (d), (e), (f), and (g) represent liver sections from rats treated with coffee, DFE, DPE, and the combination of coffee + DFE and coffee + DPE, respectively, showing marked regeneration of hepatic architecture and almost all hepatocytes become normal especially rats receiving DFE alone or combinations.

Sections of liver tissue stained with H&E (Figure 8) from normal control rat showed normal hepatocytes, portal areas, and normal architecture of hepatic lobules and blood sinusoids, while administration of CCl_4_ led to formation of foci of marked hepatic cellular degeneration with abnormal irregularly arranged hepatic lobules. Liver tissues from rats receiving coffee, DFE, DPE, or combination of coffee + DFE and coffee + DPE showed marked improvement of hepatocytes architecture of classic hepatic lobules especially in groups receiving DFE and combinations.

## 4. Discussion

Hepatic fibrosis is a pathological progression associated with excessive ECM deposition in response to chronically damaged liver tissue [1]. If hepatic fibrosis treatment was delayed, hepatic cirrhosis would be developed with associated significant morbidity and mortality. This study evaluated the protective effects of DFE or DPE against liver injury induced by repeated CCl_4_ administration in rats. The underlying mechanisms such as antioxidant action, inactivation of HSCs, and controlling fibrogenic, inflammatory and angiogenic factors were investigated. Extensive data demonstrated the hepatoprotective effects of coffee against liver fibrosis, cirrhosis, and hepatocellular carcinoma [10, 33–41]. Therefore, the hepatoprotective effects of DFE or DPE were compared to those obtained by coffee. In addition, the possible synergistic effects of the combination of DFE or DPE with coffee were also investigated.

One of the most sensitive indicators of hepatocyte injury is the release of intracellular enzymes, such as transaminases (ALT and AST), LDH, ALP, and GGT in the circulation. The enhanced activities of these enzymes are indicative of cellular leakage and loss of the functional integrity of the cell membranes in the liver [25]. In this study and consistent with earlier reports, CCl_4_ resulted in increased serum levels of ALT, AST, LDH, GGT, and ALP indicating liver dysfunction. In addition, serum albumin was reduced reflecting impaired synthetic capacity of the liver. Staining with H&E (Figure 8) illustrated marked changes in the overall histoarchitecture of the liver in response to CCl_4_. These functional and structural changes could be related to the toxic effects primarily via the generation of reactive oxygen species (ROS) causing damage to the various membrane components of the cell. Our results revealed that coffee, DFE, DPE, and the combination groups significantly ameliorated the deterioration in liver enzymes and albumin levels suggesting that coffee, DFE, and DPE improved liver function via restoring cell membrane integrity. Normalization of liver enzymes was associated with the healing of hepatic parenchyma and the regeneration of hepatocytes (Figure 8). Consistent with our results, coffee [35, 39–41], DFE [23, 25, 32, 46], and DPE [31, 32] were found to normalize liver enzymes and liver function in different models of hepatotoxicity. It is concluded that administration of coffee, DFE, or DPE significantly prevents death of hepatocytes, as can be revealed by reduced ALT and LDH activities. Moreover, they prevent cholestatic damage as can be seen by decreasing the levels of ALP and GGT. These effects were more pronounced by DFE and by the combination DFE + coffee and coffee + DPE compared to coffee alone.

Oxidative stress caused by chemicals is known to play an important causative and aggravating role in liver fibrosis. Hepatotoxicity by CCl_4_ is believed to result from CCl_4_ metabolism by cytochrome P-450 to the trichloromethyl (CCl3^∙^) and trichloromethylperoxyl (OOCCl3^∙^) radicals which can directly damage the plasma membrane, initiate the peroxidation of lipids, cause the deformation and necrosis of liver cells, and activate HSCs [47]. Free radicals induced by CCl_4_ also lead to impaired antioxidant defense system either enzymatic, such as SOD, GR, and GPx, or nonenzymatic such as GSH. MDA is one of the end products of polyunsaturated fatty acids peroxidation, and its tissue level can reflect the extent of lipid peroxidation. This study showed that the MDA content was significantly increased in the liver of CCl_4_-intoxicated rats indicating lipid oxidation that leads to tissue injury. GSH constitutes the first line of defense against free radicals. SOD is an extremely effective defense enzyme that converts superoxide anions into hydrogen peroxide (H_2_O_2_). GPx metabolises H_2_O_2_ and hydroperoxides to nontoxic products and stops the chain reaction of lipid peroxidation by removing lipid hydroperoxides from the cell membrane [48]. GR is a cytosolic hepatic enzyme that is involved in the detoxification of xenobiotics by their conjugation with GSH [49]. In this study, a significant depletion of GSH, SOD, and GPx and a significant elevation in the activity of GR were observed in CCl_4_-intoxicated rats. The decrease of GSH is probably related to a reduced synthesis of this tripeptide by the diseased liver or may be associated with its rapid consuming for scavenging ROS and free radicals in fibrotic liver. The enhanced GR activity may be explained by the increased production of ROS which must be actively scavenged by GSH, resulting in the formation of GSSG, the oxidized form of GSH, which is rapidly converted to its reduced form (GSH) by GR activity [50]. The activities of the enzymatic antioxidants SOD and GPx are reduced by lipid peroxides or ROS, which results in decreased activities of these enzymes. In addition, the liver is the main organ in the metabolism and homeostasis of selenium in the body and because GPx is a selenoprotein which is predominantly synthesized and secreted by the liver [50] so, we observed a decrease of GPx activity in CCl_4_-intoxicated rats compared with normal liver.

The significant reduction of MDA content and GR activity together with the enhanced levels of GSH, SOD, and GPx in rats treated with coffee, DFE, DPE, and the combination groups suggested the protection of the liver through antioxidant mechanism leading to inhibitory action on lipid peroxidation and restoring the normal oxidant/antioxidant balance of the liver and preservation of membrane integrity. These results, regarding DFE and DPE, supported the previous findings that DFE [23–25, 32, 46] or DPE [31, 32] exhibited hepatoprotective efficacy via antioxidant mechanisms. The antioxidant activity of date fruit is believed to be due to the wide range of polyphenolic compounds (p-coumaric, ferulic, sinapic acids, flavonoids, anthocyanins, phenolic acids, and procyanidins) and trace elements (selenium, copper, zinc, and manganese), in addition to vitamin C present in the date palm fruit [21–23]. Concerning coffee and similar to our findings, various experimental studies have indicated that coffee* per se* or their specific compounds contain antioxidant properties and protective effects against oxidative damage and fibrosis induced by hepatotoxicants in rats and mice [10, 35, 39–41].

Although previous studies have demonstrated the ability of the date extract to inhibit oxidative stress in the rodent's liver, no studies investigated the antifibrotic effects of DFE or DPE via inhibiting HSCs activation, suppression of the fibrotic, inflammatory, or angiogenic markers. Therefore, in the present study we investigated the effect of DFE or DPE on the inflammatory mediators including TNF-*α*, IL-1*β*, and IL-6, fibrotic markers including *α*-SMA (as marker of HSCs activation), hydroxyproline, collagen deposition, and TGF-*β*1, and angiogenic markers including CD31, VEGF, and VEGFR-1.

Hepatocellular injury usually leads to inflammation that is often caused by a rise in free radicals generated by various endo- and exogenous compounds processed in the liver. Upon persistence of oxidative insults in the liver, the damage from free radicals increases resulting in inflammation, activation of HSCs, and the formation of scar tissue [51]. Our data revealed elevated levels of the inflammatory cytokines including TNF-*α*, IL-6, and IL-1*β* in CCl_4_-hepatic tissues which could be related to the increased free radicals production that promote increased expression of proinflammatory mediators. Elevated levels of TNF-*α*, IL-6, and IL-1 *β* were also previously reported in liver fibrosis in rats [52–55]. The inflammatory response in our study was significantly suppressed by treatment with coffee, DFE, or DPE and by the combinations. Our results supported previous data that coffee suppressed several gene expression of TNF, IL-6, and IL-1*β* in liver tissues [10, 39, 56]. In contrast to coffee, no clear data are available concerning the anti-inflammatory effects of DFE or DPE on liver fibrosis. Our study suggests the anti-inflammatory effects of DFE or DPE as a mechanistic tool for its hepatoprotective effect through reducing the production of TNF-*α*, IL-6, and IL-1*β* in the intoxicated liver. The anti-inflammatory effect of dates could be attributed to the polyphenolic compounds that are demonstrated by its ability to inhibit the production of nitric oxide and TNF-*α* [57]. The antioxidant properties of dates and coffee could also contribute to their anti-inflammatory effects. The reduction in TNF-*α* and IL-1*β* levels was more prominent in DFE-treated rats and in the combination groups than in coffee alone or DPE alone treated groups suggesting the superior effect of DFE.

After that we investigated the effect of DFE and DPE on fibrotic markers. Our study elucidated another mechanism by which DFE or DPE could influence liver fibrosis, that is, via reducing the fibrotic markers and inactivation of HSCs that are considered as a key target in antifibrotic therapy [58, 59]. In the present study, liver fibrosis was indicated by the significant high hepatic levels of Hyp and by Masson trichome staining that showed increased collagen deposition in liver of CCl_4_-treated rats. Our results revealed also increased levels and expression of TGF-*β*1 and *α*-SMA in CCl_4_-intoxicated rats indicating HSCs activation and liver fibrosis. The key factors involved in the activation of HSC could be divided into mitogenic cytokines (which stimulate HSC proliferation) such as TGF-*β*, platelet-derived growth factor, IL-1, and TNF-*α*, and fibrogenic cytokines (which induce synthesis of matrix protein) like TGF-*β* and IL-6 [60]. These cytokines which were significantly overproduced in fibrotic liver in our study cause the HSCs activation towards myofibroblasts-like phenotype being characterized by overproduction of collagen and increased expression of *α*-SMA, which is the hallmark for activated HSCs [61]. Moreover, the excessive free radicals produced by toxic chemicals directly or indirectly contribute to the induction of TGF-*β*1 with subsequent stimulation of HSCs [61]. In addition to its role as one of most important promoting factors for HSCs activation [5], a large body of evidence shows that the liver fibrogenic response is highly regulated by TGF-*β*, because it alters the normal balance between ECM synthesis and degradation leading to accumulation of matrix components [62]. Moreover, Dooley and Ten Dijke [63] demonstrated that TGF-*β*1 contributes to fibrogenesis through inflammation. Thus prevention of TGF-*β*1 secretion is also an important target in antifibrotic therapy.

Data from our study confirmed the previous reports that coffee could reduce CCl_4_-induced liver fibrosis via reducing collagen deposition and TGF-*β*1 expression [10, 35, 39, 41, 64] and through mitigating the expression of *α*-SMA [10, 39, 65] in rat's hepatic tissue reflecting its role in preventing HSCs activation and fibrosis. Unlike coffee, there are no data about the effect of DFE and DPE on fibrotic markers and HSCs activation. Our study revealed for the first time that DFE, DPE, and their combination with coffee effectively protect liver through extenuating collagen deposition, reducing TGF-*β*1 production and suppression of *α*-SMA expression in hepatocytes. The reduction in TGF-*β*1, *α*-SMA, collagen deposition, and Hyp content was more prominent in the groups treated with DFE alone and with the combination of coffee + DFE or coffee + DPE. Collectively, our data provide strong evidence that inhibiting the release of proinflammatory mediators with the subsequent suppression of TGF-*β*1 production and inactivation of HSCs may be possible underlying mechanisms for the antifibrotic effects of the DFE and DPE in the liver.

VEGF is the central proangiogenic factor during chronic liver injury and may act as a fibrogenic during the development of liver cirrhosis. It results in changes in liver vascular architecture, increased vascular resistance, portal hypertension, decreased parenchymal perfusion, and finally triggering fibrogenic progression towards the end-point of cirrhosis. VEGF triggers the proangiogenic activity mainly by binding to two high-affinity tyrosine kinase receptors, VEGFR-1 and VEGFR-2 [12, 13, 66]. Consistent with previous reports [6–11, 14], we also found that hepatic VEGF and VEGFR-1 were upregulated in our model of chronic CCl_4_-induced liver injury. Our results therefore confirmed that the VEGF signaling pathway may trigger the microvascular proliferation associated with liver fibrogenesis, thereby contributing to the remodeling of liver architecture. It has been found that inhibition of either VEGFR-1 or VEGFR-2 significantly attenuated liver fibrogenesis accompanied by angiogenesis suppression [6]. Treatment of coffee, DFE, DPE, and the combination significantly decreased the intrahepatic expression of VEGF and VEGFR-1 in CCl_4_-treated fibrotic rats. These data revealed that the antifibrotic effect of DFE and DPE could act through the inhibition of liver fibrosis-associated angiogenesis. Coffee was also previously reported to inhibit the expression of VEGF in CCl_4_-injured liver [10]. Activated HSCs express VEGF and VEGF receptors after CCl_4_ treatment reflecting the role of HSCs in angiogenesis [11, 14, 67] and suggesting that, following activation and phenotypical modulation, HSCs tend to acquire a generic proangiogenic phenotype. Treatment with DFE and DPE mitigated the expression of *α*-SMA and this result suggests that DFE and DPE inhibition of HSC activation may be involved in fibrosis-associated angiogenesis. Moreover, inflammatory cell infiltration has often been linked to angiogenesis [68, 69] through activation of HSCs. Inflammation-associated angiogenesis might contribute to the initiation of liver fibrosis and to the progression from fibrosis into cirrhosis and finally into HCC [66]. DFE and DPE significantly lowered the hepatic levels of proinflammatory mediators including TNF-*α*, IL-6, and IL-1*β* suggesting that the anti-inflammatory effects of DFE and DPE may also contribute to their antiangiogenetic properties and consequently antifibrotic effects. Antiangiogenic effects of dates could be also attributed to their content of selenium which exhibit antiangiogenic effects in tumor tissue [70]. The antiangiogenic effect of coffee, DFE, and DPE was confirmed by detecting hepatic expression of the CD31, also known as platelet endothelial cell adhesion molecule-1 (PECAM-1). The expression of CD31 is a hallmark of capillarization [71]. As expected, increased expression of CD31 was demonstrated in CCl_4_-fibrotic liver in our study and in previous studies [72–74] confirming the formation of new blood vessels. Compared to CCl_4_-treated rats, coffee, DFE, and DPE treatment markedly decreased CD31 expression particularly in DFE and combination groups which support their antifibrotic effects through the antiangiogenic properties.

## 5. Conclusion

As for possible antifibrotic mechanisms in rat's liver, our results confirmed the antioxidant properties of date fruit (either flesh or pits) and revealed for the first time that these effects are associated with the protection of hepatocytes via anti-inflammatory mechanisms, inactivation of HSCs, and downregulation of the fibrogenic cytokine TGF-*β* and the angiogenic factors VEGF, VEGFR-1, and CD31. Our results also support the earlier findings suggesting a beneficial effect of coffee on the liver. We concluded that the antifibrotic action of DFE alone and of the combination of DFE + coffee and DPE + coffee may be superior to that of coffee alone.

## Figures and Tables

**Table 1:** Effect of date flesh extract (DFE), date pits extract (DPE), coffee, and the combination groups on liver function markers in CCl_4_-intoxicated rats.

Group	ALT (U/mL)	AST (U/mL)	LDH (U/mL)	ALP (U/mL)	GGT (U/mL)	Albumin (g/dL)
Normal control	88.7 ± 4.6	280.6 ± 7.3	1264 ± 95	77.8 ± 6	21.2 ± 0.39	4.1 ± 0.17
CCl_4_	255^a∗∗∗^ ± 21.8	409^a∗∗∗^ ± 26.8	1735^a∗∗^ ± 126	175.8^a∗∗∗^ ± 13	41.4^a∗∗∗^ ± 1.4	2.9^a∗∗∗^ ± 0.26
Coffee	124^b∗∗∗^ ± 4.2	345.8^b∗^ ± 11.9	1106^b∗∗∗^ ± 50	121^b∗^ ± 12.6	34.3^b∗^ ± 1.13	3.8^b∗∗∗^ ± 0.08
DFE	82.4^b∗∗∗c∗^ ± 4.5	321.8^b∗∗^ ± 12.4	1096^b∗∗∗^ ± 66	79.3^b∗∗∗c∗^ ± 6.3	25.5^b∗∗∗c∗^ ± 1.3	4.01^b∗∗∗^ ± 0.12
DPE	111.4^b∗∗∗^ ± 4.0	343^b∗^ ± 10.8	1207^b∗∗∗^ ± 83	93^b∗∗∗^ ± 12.7	28.4^b∗∗∗^ ± 1.4	3.7^b∗∗∗^ ± 0.1
Coffee + DFE	74.1^b∗∗∗c∗^ ± 3.5	326.2^b∗∗^ ± 15	1088^b∗∗∗^ ± 31	81.3^b∗∗∗c∗^ ± 7.8	23.8^b∗∗∗c∗^ ± 1.77	3.9^b∗∗∗^ ± 0.09
Coffee + DPE	73^b∗∗∗c∗^ ± 6.5	340.1^b∗^ ± 9.7	1208^b∗∗∗^ ± 70	92.5^b∗∗∗^ ± 5	27.6^b∗∗∗^ ± 1.8	3.8^b∗∗∗^ ± 0.07

^a^Significantly different from normal control group; ^b^significantly different from CCl_4_-treated group; ^c^significantly different from coffee-treated group. ^***^P < 0.001, ^**^P < 0.01, ^*^P < 0.05.
